# ‘Controversies on risk factors, diagnosis and treatment of male infertility: Is there an end?’

**DOI:** 10.1080/20905998.2025.2515742

**Published:** 2025-06-08

**Authors:** Ramadan Saleh, Taymour Mostafa, Selahittin Çayan, Rupin Shah, Ashok Agarwal

**Affiliations:** Department of Dermatology, Venereology and Andrology, Faculty of Medicine, Sohag University, Sohag, Egypt; Ajyal IVF Center, Ajyal Hospital, Sohag, Egypt; Global Andrology Forum, Global Andrology Foundation, Moreland Hills, OH, USA; Department of Andrology, Sexology & STIs, Faculty of Medicine, Cairo University, Cairo, Egypt; Department of Urology, Andrology Section, School of Medicine, University of Mersin, Mersin, Turkey; Division of Andrology, Department of Urology, Lilavati Hospital and Research Centre, Mumbai, India; Cleveland Clinic Foundation, Cleveland, OH, USA

## Foreword

Infertility remains a significant global health concern, affecting approximately 15% of couples worldwide and often leading to profound psychosocial distress and diminished quality-of-life. Notably, male factor infertility contributes to nearly 50% of all cases. Despite advances in understanding male infertility pathogenesis, diagnostic innovations, and therapeutic developments, several clinical debates persist. Crucially, a substantial proportion of men remain classified as having idiopathic infertility, lacking an identifiable etiology.

This special issue addresses several of the most pressing controversial topics in male infertility, with a focus on lifestyle and environmental factors that affect male fertility. These aspects are explored through several key articles. Rocco et al. examine the impact of obesity on male reproductive health [1], while Kavoussi & Kavoussi analyze the potential effects of mobile phones and laptops on sperm function [2]. Kumar et al. discuss the reproductive consequences of testosterone abuse [3]. A comprehensive review by Bocu et al. further investigates the biological and ecological mechanisms by which environmental exposures affect spermatogenesis and sperm quality, offering potential lifestyle-based interventions [4].

This issue also tackles diagnostic challenges in male infertility, such as male accessory gland infections by Dutta et al. [5] and testicular microlithiasis, a frequently incidental finding during scrotal ultrasound in infertile men by Yilmaz et al. [6]. Additionally, Boeri et al. provide fresh insights into new pathophysiological mechanisms shaping the management of idiopathic male infertility [7].

Further perspectives are presented on specific conditions affecting infertile men, including varicocele cases with normal semen parameters but elevated sperm DNA fragmentation by Almekaty et al. [8], as well as cryptozoospermia and the ongoing debate on optimal sperm retrieval strategies by Japari et al. [9].

Bringing together a diverse, internationally recognized group of authors, this issue presents a multidisciplinary and evidence-based evaluation of current challenges in male infertility. By encouraging collaboration across specialties, these contributions aim to reduce clinical uncertainty, improve treatment approaches, and ultimately enhance reproductive outcomes for infertile men. This collection offers critical insights and serves as a valuable resource for both clinicians and researchers committed to advancing the field of andrology.
